# Novel traction device for endoscopic submucosal dissection: a rotatable transparent cap, an additional channel, and a traction wire

**DOI:** 10.1055/a-2222-6958

**Published:** 2024-01-23

**Authors:** Xi Chen, Zhenqun Lin, Xiangqing Li, Xiaoning Yang, Wenjuan Liu, Zehui Huang, Shuntian Cai

**Affiliations:** 1 Gastroenterology, Zhangzhou Traditional Chinese Medicine Hospital, Zhangzhou, China; 2 47858 Gastroenterology, Fujian University of Traditional Chinese Medicine, Fuzhou, China; 3 Intelligent Biomedical Robot Lab, College of Artificial Intelligence and Big Data for Medical Sciences, Shandong First Medical University & Shandong Academy of Medical Sciences, Jinan, China; 4 Gastroenterology, Zhongshan Hospital of Xiamen University, School of Medicine, Xiamen University, Xiamen, China; 5 Xiamen Key Laboratory of Intestinal Microbiome and Human Health, Zhongshan Hospital of Xiamen University, School of Medicine, Xiamen University, Xiamen, China; 6 47858 Fujian University of Traditional Chinese Medicine, Fuzhou, China; 7 Endocrinology, Zhangzhou Traditional Chinese Medicine Hospital, Zhangzhou, China; 8 Zhangzhou Traditional Chinese Medicine Hospital, Zhangzhou, China; 9 Xiamen Key Laboratory of Intestinal Microbiome and Human Health, Zhongshan Hospital of Xiamen University, Xiamen, China

Traditionally, endoscopic procedures have been limited to a single–channel device, making it difficult for endoscopists to perform endoscopic submucosal dissection (ESD). As a result, several solutions have been developed, each with its own advantages and disadvantages, including traction devices 1 2 , double-channel endoscopes 3 , laparoscopy–endoscopy combined surgery 4 , and surgical endoscopic robots 5 . To address these challenges, we fabricated a rotatable transparent cap with a fixed channel that can accommodate endoscopic surgical instruments.

The traction device consists of three components: a rotatable transparent cap, an additional channel, and a traction wire ( Fig. 1 ). The additional channel and traction wire are connected to a rotatable ring on the transparent cap. By pulling the traction wire, the additional channel can be rotated around the transparent cap ( Fig. 1 ).

**Fig. 1:**
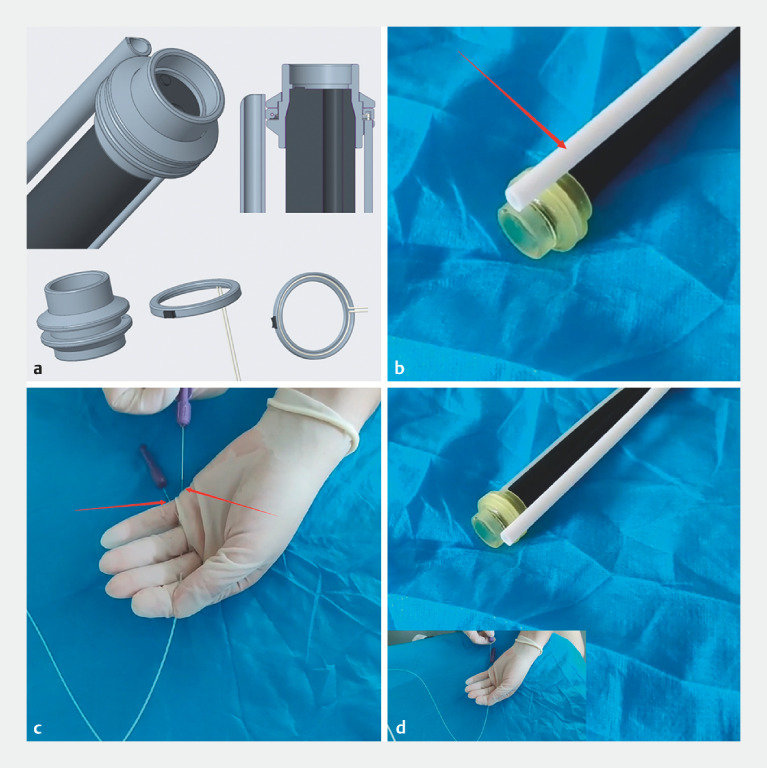
Structure and functionality of the rotatable transparent cap device (Micro-Tech, Nanjing, China). **a** The rotatable transparent cap unit consists of a transparent cap with a rotatable ring in the middle section. A nylon wire threaded through a channel in the rotatable ring is fixed at its midpoint to the ring. **b** Auxiliary channel fixed to the rotatable ring. **c** Ends of the nylon wire that is connected to the ring and additional channel. Both ends of the wire can be used to pull the ring, rotating it. **d** The wire can be pulled to rotate the auxiliary channel on the ring around the transparent cap, enabling precise control.

During ESD, foreign-body forceps are inserted into the additional channel to clamp and push the tumor to the distal end, acting as a traction device. During the operation, the position of the additional channel can be controlled dynamically by pulling the traction wire. To assess the efficacy of this device, we performed ESD in four directions: downward, upward, leftward, and rightward. The results demonstrated that this rotatable transparent cap device enables dynamic rotation of the foreign-body forceps in various directions to apply traction ( Video 1 ). This not only decreases the complexity of the procedure and the associated risks, but also leads to a significant reduction in operation time and incidence of complications.

Structural features, functionality, and application of the novel traction device for endoscopic submucosal dissection (ESD) using an isolated pig stomach.Video 1

In conclusion, this device exhibits promise for facilitating traction during ESD, simplifying the procedure and eliminating the need for specialized endoscopic equipment.

Endoscopy_UCTN_Code_TTT_1AQ_2AD

## Notes

Endoscopy E-Videos https://eref.thieme.de/e-videos
*E-Videos* is an open access online section of the journal *Endoscopy* , reporting on interesting cases and new techniques in gastroenterological endoscopy. All papers include a high-quality video and are published with a Creative Commons CC-BY license. Endoscopy E-Videos qualify for HINARI discounts and waivers and eligibility is automatically checked during the submission process. We grant 100% waivers to articles whose corresponding authors are based in Group A countries and 50% waivers to those who are based in Group B countries as classified by Research4Life (see: https://www.research4life.org/access/eligibility/ ). This section has its own submission website at https://mc.manuscriptcentral.com/e-videos .

## References

[1] Li D Xie J Hong D Clip and dental floss traction-assisted endoscopic mucosal resection for early carcinoma of the duodenal papilla Endoscopy 2023 55 E440 E441 10.1055/a-2011-5788 36796445 PMC9935074

[2] Liu J Fang N Traction by dental floss loop for adequate submucosal dissection depth in a rectal neuroendocrine tumor Endoscopy 2023 55 E326 E327 10.1055/a-1974-9297 36513110 PMC9833942

[3] de Melo SW Jr Cleveland P Raimondo M Endoscopic mucosal resection with the grasp-and-snare technique through a double-channel endoscope in humans Gastrointest Endosc 2011 73 349 352 21295646 10.1016/j.gie.2010.10.030

[4] Yorimitsu N Oyama T Takahashi A Laparoscopy and endoscopy cooperative surgery is a safe and effective novel treatment for duodenal neuroendocrine tumor G1 Endoscopy 2020 52 E68 E70 10.1055/a-0999-5172 31529442

[5] Ho KY Phee SJ Shabbir A Endoscopic submucosal dissection of gastric lesions by using a Master and Slave Transluminal Endoscopic Robot (MASTER) Gastrointest Endosc 2010 72 593 599 10.1016/j.gie.2010.04.009 20646698

